# Cerium Oxide Nanoparticles Alleviate Hepatic Fibrosis Phenotypes In Vitro

**DOI:** 10.3390/ijms222111777

**Published:** 2021-10-29

**Authors:** Adrian Boey, Shu Qing Leong, Sayali Bhave, Han Kiat Ho

**Affiliations:** Department of Pharmacy, Faculty of Science, National University of Singapore, 18 Science Drive 4, Singapore 117559, Singapore; phabhka@nus.edu.sg (A.B.); leongshuqing@gmail.com (S.Q.L.); sayali_bhave@u.nus.edu (S.B.)

**Keywords:** liver fibrosis, cerium oxide nanoparticles, nanoparticles

## Abstract

Exposure to metallic nanoparticles (NPs) can result in inadvertent NP accumulation in body tissues. While their subsequent cellular interactions can lead to unintended consequences and are generally regarded as detrimental for health, they can on occasion mediate biologically beneficial effects. Among NPs, cerium oxide nanoparticles (CeO_2_ NP) possess strong antioxidant properties and have shown to alleviate certain pathological conditions. Herein, we show that the presence of cubic 25 nm CeO_2_ NP was able to reduce TGF-β-mediated activation in the cultured hepatic stellate cell line LX2 by reducing oxidative stress levels and TGF-β-mediated signalling. These cells displayed reduced classical liver fibrosis phenotypes, such as diminished fibrogenesis, altered matrix degradation, decreased cell motility, modified contractability and potentially lowered autophagy. These findings demonstrate that CeO_2_ NP may be able to ameliorate hepatic fibrosis and suggest a possible therapeutic pathway for an otherwise difficult-to-treat condition.

## 1. Introduction

Chronic liver diseases arising from differing aetiologies often follow a defined pathway of initial fatty liver development together with increased liver fibrosis, leading to increased inflammation (hepatitis), exaggerated fibrosis (cirrhosis), and potentially hepatocellular carcinoma and liver failure. Liver fibrosis is therefore a crucial convergent point leading to several liver pathologies and represents an important opportunity to arrest or mitigate these syndromes.

Hepatic fibrosis is mediated primarily through hepatic stellate cells (HSCs). Upon hepatic injury, a complicated signalling cascade involving multiple cell types, such as hepatocytes, Kupffer cells and sinusoidal endothelial cells, results in the release of activation signals, such as tumour necrosis factor alpha (TNF-α) and transforming growth factor beta (TGF-β) [1,2]. Receipt of these signals transforms quiescent HSCs into activated myofibroblasts, which gain motility and migrate to wound sites to begin extruding extracellular matrix (ECM) materials as part of the wound-healing response. Curbing HSC activation is thus considered a prime target for liver fibrosis intervention. While numerous strategies have been proposed, there has not been any solution successfully brought to market thus far [3,4].

Metallic nanoparticles (NPs) have become increasingly ubiquitous in our environments. These tiny particles can assert disproportionately greater biological effects compared to their bulk analogues due to their cumulatively larger surface area [5,6]. Their small size allows them to easily enter the body primarily through ingestion and inhalation, and once inside, are able to penetrate into visceral tissues [6]. Numerous papers have highlighted the dangers of NP accumulation within organs; yet, at the same time, other studies have identified potentially beneficial outcomes from NP presence in specific tissues [5]. It may be that whether a NP has a net positive or negative effect depends on multiple factors, including the identity and composition of the specific NP, its size and shape and the cell types that it interacts with [5].

Cerium oxide is commonly used in manufacturing processes for producing items, such as vehicle catalytic converters, fuel cells and biomedical devices. Chemically, cerium oxide nanoparticles (CeO_2_ NP) have shown strong antioxidant effects due to their specific elemental properties, which allow for the alleviation of some pathologies [7]. Cerium ions on the surface of CeO_2_ NP are able to oscillate between two valence states (Ce^3+^ and Ce^4+^). The Ce^3+^ ions can couple with reactive oxygen or nitrogen species to reduce them to peroxides and nitrates, while itself oxidizing into the Ce^4+^ valence state, similar to the functions of the superoxide dismutase enzyme (Sod) [8,9]. The subsequent reduction from Ce^4+^ back to Ce^3+^ can oxidise hydrogen peroxide into molecular oxygen, thus mimicking the action of the catalase enzyme [9]. Such redox cycling results in the lowering of cellular ROS/RNS and hydrogen peroxide levels. In addition, CeO_2_ NP have also demonstrated several other forms of activity remarkably similar to other biological enzymes [10]. Numerous studies have pointed out that their strong redox properties confer protective effects against a host of conditions, such as neurological conditions [11,12], ischemic stroke [13] and retinal diseases [14], amongst others (recently reviewed in [15]).

The liver is the primary site of accumulation for NPs exposed via the oral route, and CeO_2_ NP have been observed to accumulate and persist in hepatic tissues. One study examining CeO_2_ NP accumulation in rats that were exposed via inhalation assays found that CeO_2_ NP were able to penetrate the lung barrier and access interior tissues, and even so, the liver showed the second highest tissue CeO_2_ NP concentration after the lungs [16]. Yokel and colleagues tested several high dosages of CeO_2_ NP and showed highest accumulation in the liver and the spleen [17]. Numerous other papers have found the liver to be one of the primary sites of CeO_2_ NP accumulation in vivo [18,19,20,21,22].

Coupling this natural accumulation with the strong redox potential of CeO_2_ does present some latent potential as therapeutics against liver pathologies. Firstly, CeO_2_ NP have been shown to alleviate effects of xenobiotic-induced liver injuries, including diethylnitrosamine, acetaminophen and doxorubicin [23,24,25,26]. Secondly, several studies have reported using CeO_2_ NP to treat non-alcoholic fatty liver disease (NAFLD) through exploiting the high redox capacities of CeO_2_ NP to counter characteristically elevated reactive oxygen species (ROS) levels in NAFLD. CeO_2_ NP treatments were found to reduce NAFLD markers, such as steatosis, inflammation, portal hypertension, hepatic lipid droplet size and content, fatty acid concentrations and compositions and the expression of NAFLD-signalling pathways [27,28,29]. Furthermore, CeO_2_ NP have also been found to have therapeutic benefit for hepatocellular carcinoma [30].

However, the evidence for CeO_2_ NP being effective against hepatic fibrosis is less clear. CeO_2_ NP (spherical, 4–20 nm) tested on rats with carbon tetrachloride (CCl_4_) induced hepatic fibrosis [27]. These rats displayed reduced steatosis and portal hypertension, but significantly only showed reduced smooth muscle actin (α-SMA) expression while collagen-I (Col-I) expression remained unchanged. Hirst and coworkers also investigated CeO_2_ NP (3–5 nm) and CCl_4_-induced liver fibrosis in mice and found that CeO_2_ NP reduced oxidative stress damage markers to levels comparable to *N*-acetylcysteine [19]. Other NP types, such as TiO_2_ NP, silicon dioxide NP [31], zinc oxide NP [32] and selenium NP [33], have previously been shown to inhibit liver fibrosis, demonstrating that NPs are indeed capable of attenuating the fibrotic liver phenotype.

We therefore sought to investigate liver fibrosis in the human cultured HSC cell line LX2 to confirm if CeO_2_ NP treatment was able to reduce fibrosis symptoms in vitro. Our results show that cubic 25 nm CeO_2_ NP were able to significantly suppress fibrosis in TGF-β-activated LX2 cells through the reduction of oxidative stress and TGF-β signalling, thus leading to a reduction in HSC activation and ultimately hepatic fibrosis. This suggests a possible role for CeO_2_ NP for treating liver fibrosis.

## 2. Results

### 2.1. Characterizing Cerium Oxide Nanoparticles Used in This Study

**Figure 1:**
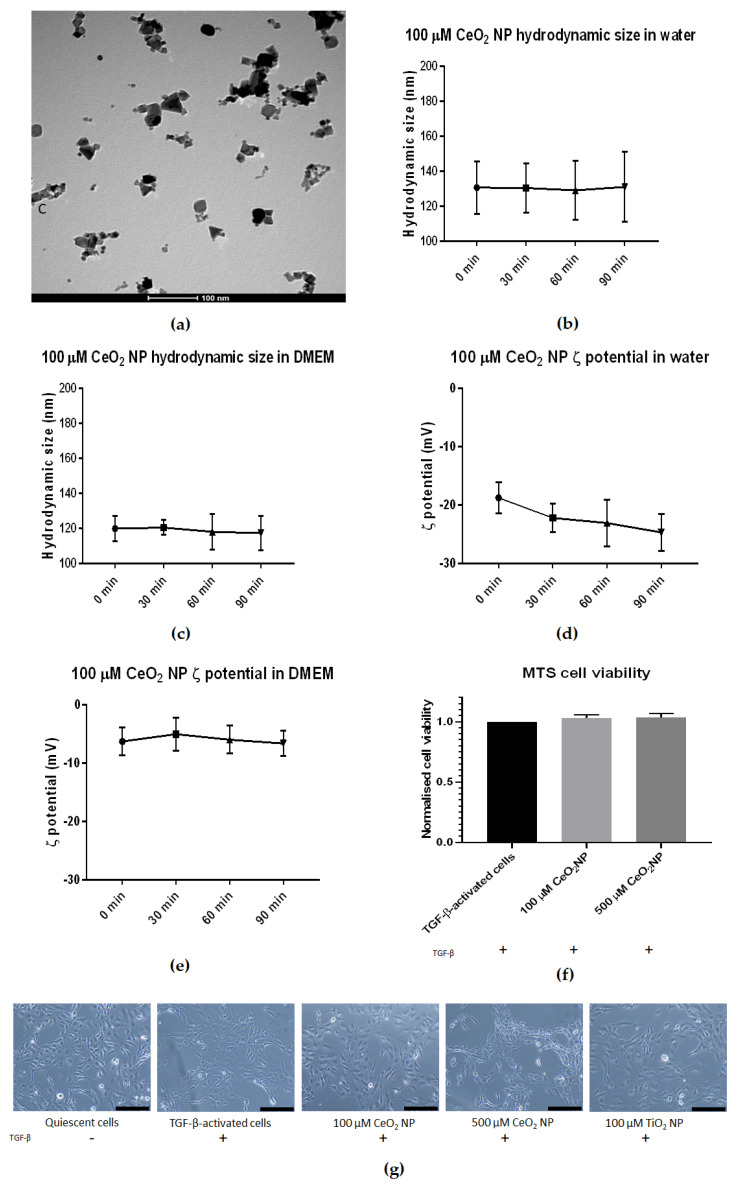
Characterisation of CeO_2_ NP used in this study. (**a**) TEM analysis revealed CeO_2_ NP to be cubic-shaped and approximately 25 nm in diameter. (**b**) DLS analysis revealed CeO_2_ NP hydrodynamic size in water to be stable over 90 min at approximately 130 nm (*n* = 3). (**c**) CeO_2_ NP hydrodynamic size in 1% DMEM showed a slightly smaller size at approximately 120 nm and was also stable over 90 min (*n* = 3). (**d**) CeO_2_ NP in water showed a downward trend in ζ potential (*n* = 3). (**e**) CeO_2_ NP ζ potential in 1% DMEM showed more stable ζ potential (*n* = 3). (**f**) 100 µM and 500 µM CeO_2_ NP showed similar cell viability as TGF-β-treated control cells when measured using MTS assay (*n* = 3). (**g**) Light microscope images taken at 10× magnification of quiescent cells, TGF-β-activated cells, 100 µM CeO_2_ NP, 500 µM CeO_2_ NP and TiO_2_ NP-treated cells (*n* = 3). Scale bar represents 200 µm. Error bars represent standard error of the mean (SEM); one-way analysis of variance (ANOVA) was used to calculate significance.

The CeO_2_ NP used in this study were first characterised using transmission electron microscopy (TEM), dynamic light scattering (DLS) and zeta potential (ζ potential) measurement (Figure 1). TEM images confirmed the CeO_2_ NP to be cubic-shaped and approximately 25 nm in diameter (Figure 1a). Dynamic light scattering found that the hydrodynamic size of CeO_2_ NP in water and Dulbecco’s Modified Eagle’s Medium (DMEM) was approximately 120 nm and 160 nm in diameter, respectively (Figure 1b,c). Crucially, their DLS sizes showed little change in size when measured at 30-min intervals over 90 min in both water and 1% fetal bovine serum (FBS) DMEM (Figure 1b,c). Finally, CeO_2_ NP resuspended in water showed negative ζ potential charges over time (−23.3 mV after 90 min) (Figure 1d), while CeO_2_ NP in 1% FBS DMEM showed higher negative ζ potential than those resuspended in water (−6.63 mV after 90 min) (Figure 1e).

We next performed cell viability assays to prove that CeO_2_ NP were not toxic to LX2 cells at 100 and 500 µM concentrations. We used 3-(4,5-dimethylthiazol-2-yl)-5-(3-carboxymethoxyphenyl)-2-(4-sulfophenyl)-2H-tetrazolium] (MTS) assays to assess the viability of TGF-β-activated cells after 24 h of co-incubation with 100 and 500 µM CeO_2_ NP (Figure 1f). Cell viability was not significantly different compared to TGF-β-activated controls, demonstrating that CeO_2_ NP presence was not detrimental for the cells at the concentrations used.

Lastly, light microscope images at 10× magnification were taken of LX2 cells after 24 h of incubation under different treatment conditions (quiescent cells, TGF-β-activated cells, TGF-β-activated cells with 100 µM CeO_2_ NP, TGF-β-activated cells with 500 µM CeO_2_ NP and TGF-β-activated cells with 100 µM titanium dioxide nanoparticles (TiO_2_ NP)). The morphology of NP/TGF-β-treated cells showed no obvious difference compared to TGF-β-activated cells, suggesting that these NPs did not exert any detrimental effects on cell morphology.

### 2.2. Cerium Oxide Nanoparticles Are Able to Reduce Fibrogenesis Marker Expression

**Figure 2:**
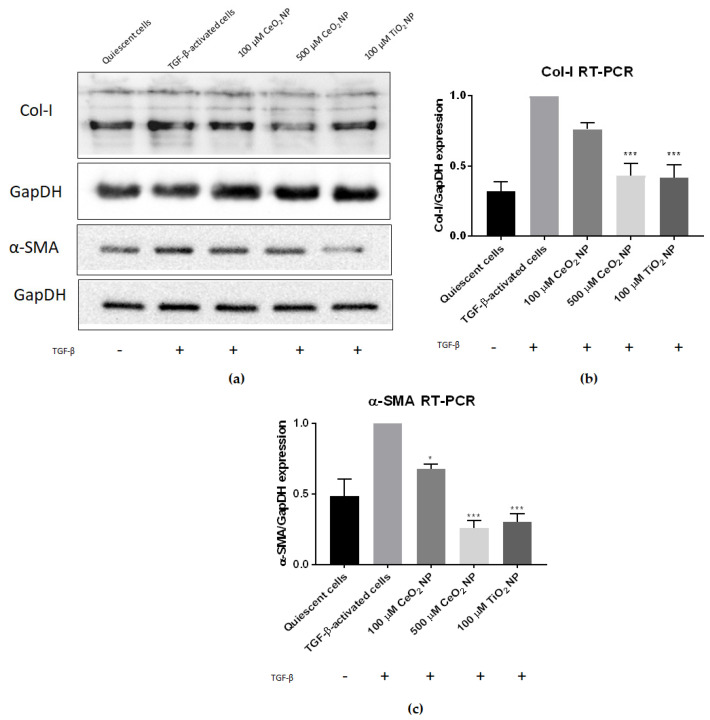
CeO_2_ NP reduce fibrogenesis in TGF-β-activated cells. (**a**) Immunoblot analysis showed a dose-dependent reduction in both Col-I and α-SMA protein expression (*n* = 4). (**b**) RT-PCR analysis found Col-I expression in 500 µM CeO_2_ NP and TiO_2_ NP-treated cells to be significantly reduced (*n* = 3). (**c**) RT-PCR analysis found α-SMA expression in 100 µM CeO_2_ NP, 500 µM CeO_2_ NP and TiO_2_ NP-treated cells to be significantly reduced (*n* = 3). Error bars represent SEM; ANOVA was used to calculate significance. * *p* < 0.05, *** *p* < 0.001.

To determine if CeO_2_ NP are able to reduce fibrosis, we first examined the expression of fibrotic marker genes collagen-I and smooth muscle actin using real-time PCR (RT-PCR) and immunoblotting in TGF-β-activated cells, with or without CeO_2_ NP (Figure 2). Expression was normalised to TGF-β-activated controls to detect if CeO_2_ NP exposure reduced Col-I or α-SMA expression.

It can be seen that treating TGF-β-activated LX2 cells with CeO_2_ NP significantly reduced Col-I and α-SMA expression (Figure 2). Immunoblot images showed reduced Col-I and α-SMA protein expression (Figure 2a), demonstrating that CeO_2_ NP treatment reduced fibrogenesis marker expression. RT-PCR analysis of Col-I mRNA expression showed that 100 µM CeO_2_ NP (equivalent to 17.2 µg/mL) and 500 µM CeO_2_ NP (equivalent to 86.1 µg/mL) reduced Col-I by 24% percent and 67% percent respectively compared to TGF-β-activated controls (Figure 2b). Similarly, 100 µM and 500 µM CeO_2_ NP treatment showed significant reduction of α-SMA transcript expression (32% and 74% respectively) (Figure 2c). Titanium dioxide nanoparticles (TiO_2_ NP) have previously been shown to possess antifibrotic capacities [31] and k9were used as an experimental positive control in this study. Agreeably, 100 µM TiO_2_ NP (equivalent to 0.799 µg/mL) were able to significantly reduce Col-I and α-SMA expression in our experiments. Noticeably, only 500 µM CeO_2_ NP were able to reduce Col-I (67% vs. 69% reduction when measured using RT-PCR) and α-SMA expression (74% vs. 69% reduction when measured using RT-PCR) to levels comparable to 100 µM TiO_2_ NP.

### 2.3. CeO_2_ NP Affect Other Classical Hallmarks of Hepatic Fibrosis

**Figure 3:**
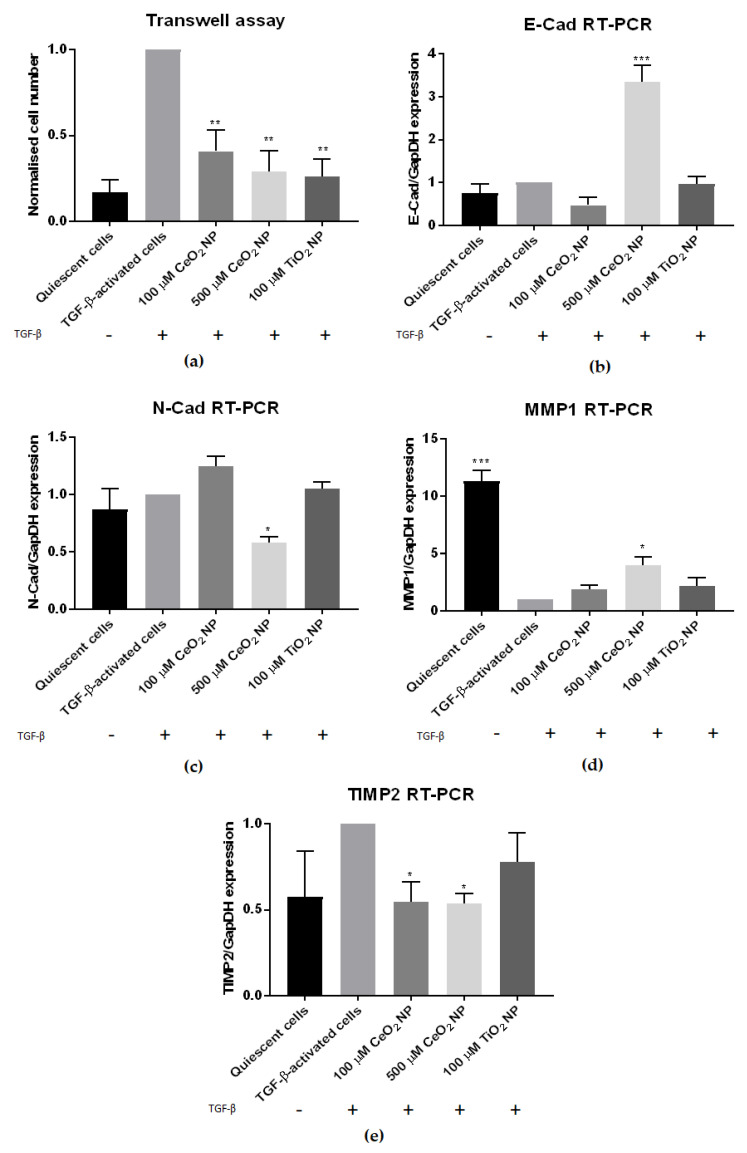
CeO_2_ NP-treated cells show reduced hepatic fibrosis hallmarks. (**a**) Transwell assay examining the cell motility of CeO_2_ NP-treated cells showed significantly reduced motility in 100 µM, 500 µM and TiO_2_-treated cells. (*n* = 3) (**b**) The 500 µM CeO_2_ NP-treated cells showed significantly elevated E-cad mRNA expression. (*n* = 3) (**c**) The 500 µM CeO_2_ NP-treated cells also had significantly reduced N-cad mRNA expression. (*n* = 3) (**d**) RT-PCR analysis showed significantly higher MMP1 expression in 500 µM CeO_2_ NP-treated cells. (*n* = 3) (**e**) TIMP2 mRNA expression was significantly downregulated in 100 µM CeO_2_ NP and 500 µM CeO_2_ NP-treated cells. (*n* = 4). Error bars represent SEM; ANOVA was used to calculate significance. * *p* < 0.05, ** *p* < 0.01, *** *p* < 0.001.

We next set out to determine if other characteristics of fibrosis are also reduced in CeO_2_ NP-treated cells. In addition to increased fibrogenesis, activated stellate cells display other characteristics, such as increased chemotaxis movement, elevated contractility and altered matrix remodelling [1]. Accordingly, TGF-β-activated LX2 cells were treated with CeO_2_ NP for 24 h before being tested for their degree of motility using a transwell assay. Figure 3a shows the total number of migrated cells counted/chamber, normalised to TGF-β-activated controls. TGF-β-activated cells retained their strong cell motility phenotype, but in contrast 100 µM, 500 µM CeO_2_ NP and TiO_2_ NP-treated cells showed significantly reduced cell motility, indicating that these cells were less motile compared to TGF-β controls.

We went on to examine contractility by means of epithelial-mesenchymal biomarkers in CeO_2_ NP-treated cells. N-cadherin (N-cad) and E-cadherin (E-cad) expression was measured in CeO_2_ NP-treated cells using RT-PCR. At 500 µM, CeO_2_ NP significantly upregulated E-cad expression and downregulated N-cad expression (Figure 3b,c).

We next sought to investigate if matrix remodelling was altered in CeO_2_ NP-treated cells through using RT-PCR to examine the expression of matrix metalloproteinase 1 (MMP1) and tissue inhibitor of matrix metalloproteinase 2 (TIMP2). MMP1 was significantly upregulated in 500 µM CeO_2_ NP-treated cells (Figure 3d), while TIMP2 was significantly downregulated in both 100 µM CeO_2_ NP and 500 µM CeO_2_ NP-treated cells (Figure 3e).

**Figure 4:**
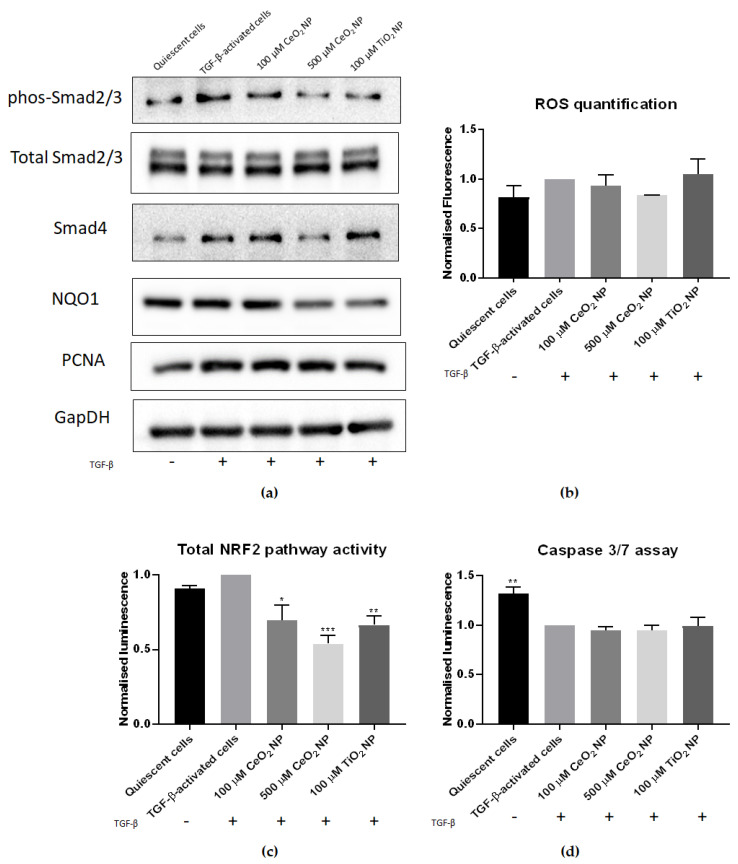
CeO_2_ NP treatment reduces hallmarks of HSC activation. (**a**) Phosphorylated Smad2/3, total Smad2/3, Smad4 and NQO1 immunoblots. Phosphorylated Smad2/3 and Smad4 protein expression was decreased in 500 µM CeO_2_ NP-treated cells. Similarly, NQO1 expression was reduced in CeO_2_ NP-treated cells. PCNA expression showed no difference between TGF-β controls and CeO_2_ NP-treated samples. (*n* = 4) (**b**) ROS levels in CeO_2_ NP-treated cells were measured using flow cytometry. It can be seen that 500 µM CeO_2_ NP reduced oxidative stress to levels comparable to quiescent cell controls. (*n* = 3) (**c**) Nrf2 pathway activity. Total Nrf2 activity was measured using an ARE-promoter assay. The 100 µM CeO_2_ NP, 500 µM CeO_2_ NP and TiO_2_ NP-treated cells all showed significantly reduced Nrf2 activity. (*n* = 3) (**d**) Caspase 3/7 activity assay. CeO_2_ NP-treated cells showed no significant difference in caspase 3/7 activity compared to TGF-β controls. However, quiescent cells showed significantly higher caspase 3/7 activity. (*n* = 3). Error bars represent SEM; ANOVA was used to calculate significance. * *p* < 0.05, ** *p* < 0.01, *** *p* < 0.001.

We investigated reactive oxygen species (ROS) levels in CeO_2_ NP-treated cells to determine if CeO_2_ NP exposure would reduce oxidative stress. We performed flow cytometry experiments using CellROX Deep Red oxidative stress dye (Figure 4b), where TGF-activated cells were treated with CeO_2_ NP and TiO_2_ NP. Their levels of oxidative stress were quantified, and results were normalised to TGF-activated cells control and tabulated (Table 1). While we observed an increase in ROS levels between quiescent and activated cells, this difference was marginal at only 0.18 (mean value of 0.82 vs. 1.0 respectively). Corroborating with the strong redox potential of CeO_2_ NP, 500 µM CeO_2_ NP-treated cells demonstrated the largest reduction in oxidative stress amongst the samples tested (mean value of 0.79). This was lower than the amount of ROS seen in quiescent cells (mean value of 0.817). Overall, a total reduction of 113.0% in ROS levels for 500 µM CeO_2_ NP was observed when quiescent cell ROS levels were used as a baseline for comparison. In comparison, TiO_2_ NP showed a mean ROS level of 1.04, signifying that TiO_2_ NP induced oxidative stress instead. Flow cytometry data charts are found in Supplementary Figure S1.

We next examined the activity of antioxidant pathways in CeO_2_ NP-treated cells by performing antioxidant response element (ARE)-driven luciferase assays. CeO_2_ NP-treated cells showed a marked dose-dependent reduction in nuclear factor erythroid 2–related factor 2 (Nrf2) activity compared to TGF-β-activated controls (Figure 4c). To confirm this, we also studied the expression of the Nrf2-responsive gene NAD(P)H quinone dehydrogenase 1 (NQO1) in CeO_2_ NP cells using immunoblotting. NQO1 expression was similarly found to be reduced (Figure 4a), suggesting a downregulation in Nrf2 activity in CeO_2_ NP-treated cells.

### 2.4. HSC Activation Phenotypes Are Reduced in CeO_2_ NP-Treated Cells

We have shown that fibrosis levels are reduced in CeO_2_ NP-treated cells. To investigate the mechanisms underlying this, we subsequently evaluated classical features of HSC activation in these cells.

We next investigated TGF-β signalling in CeO_2_ NP-treated cells. To determine if Smad-signalling activity was reduced in CeO_2_ NP-treated cells, we examined phosphorylated Smad2/3, total Smad2/3 protein, and Smad4 expression using immunoblotting. We found that CeO_2_ NP treatment downregulated Smad2/3 phosphorylation, affirming that the effector arm of TGF-β signalling was reduced in these cells (Figure 4a). To confirm this, we also examined Smad4 expression in CeO_2_ NP-treated cells. We found Smad4 expression to be reduced as well (Figure 4a), confirming that TGF-β signalling is reduced in CeO_2_ NP-treated cells.

We also examined if CeO_2_ NP-treatment affected cell proliferation. Immunoblot analysis revealed that CeO_2_ NP-treated cells showed no difference in proliferating cell nuclear antigen (PCNA) expression compared to control TGF-β-activated cells (Figure 4a), demonstrating that DNA replication was unchanged in these cells. This suggests that cell proliferation is unaffected in these cells.

The degree of apoptosis was also studied in CeO_2_ NP-treated cells. We performed caspase 3/7 activity assays, which revealed no significant differences in caspase 3 activity between TGF-β-treated control cells and both 100 µM and 500 µM CeO_2_ NP-treated cells (Figure 4d). This indicated that CeO_2_ NP treatment did not actively promote apoptosis activity in TGF-β-activated cells.

**Figure 5:**
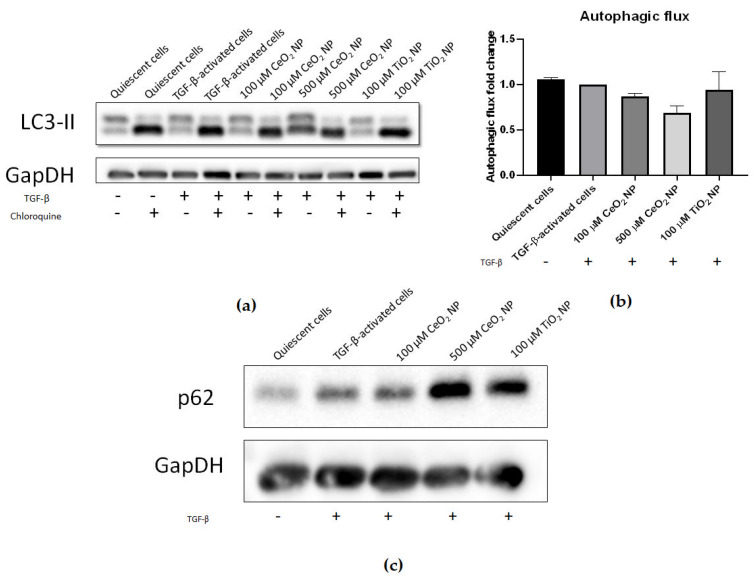
Autophagy is potentially reduced in CeO_2_ NP-treated cells. (**a**) Immunoblots of LC3-II expression in CeO_2_ NP-treated cells with and without the autophagy inhibitor chloroquine. (*n* = 4) (**b**) The 100 µM and 500 µM CeO_2_ NP showed reduced autophagic flux in a dose-dependent manner when calculated using the ratio of LC3-II expression in chloroquine-treated and chloroquine-untreated cells and measured using densitometry analysis (*n* = 4). (**c**) p62 expression was increased in 100 µM and 500 µM CeO_2_ NP-treated cells when investigated using immunoblotting (*n* = 5). Error bars represent SEM; ANOVA was used to calculate significance.

We sought to investigate if autophagy had been modified in CeO_2_ NP-treated cells through examining the expression of key autophagic markers. Microtubule-associated proteins 1A/1B light chain 3B (LC3-II) expression was compared between cells treated with and without chloroquine using immunoblotting to assess the degree of LC3-II accumulation and hence autophagic flux. It can be seen that 500 µM CeO_2_ NP treatment showed reduced autophagic flux compared to TGF-β controls (Figure 5a,b), again supporting that HSC activation is decreased in CeO_2_ NP-treated cells in a dose-dependent manner. However, this result was not found to be statistically significant. In addition, the expression of p62/sequestosome-1 (p62) was also analysed using immunoblotting. CeO_2_ NP-treated cells showed increased p62 expression in a dose-dependent manner (Figure 5c), demonstrating that CeO_2_ NP treatment was potentially able to inhibit autophagy.

## 3. Discussion

This study set out to investigate the potential of 25 nm CeO_2_ NP to treat liver fibrosis by using the immortalised human HSC cell line LX2 as an in vitro model that recapitulates the machinery of liver fibrosis [34].

We began this project by defining the physical characteristics of the CeO_2_ NP used. Aggregated NPs have been postulated to cause toxicity not seen with dispersed single particle NPs, emphasising the importance of ensuring a disperse solution. Under the conditions we used, CeO_2_ NP in both distilled water and 1% FBS DMEM solutions remained disperse when tracked using DLS. Their sizes remained stable in both solutions throughout the experiments, indicating that CeO_2_ NP did not aggregate for up to 90 min post-dispersion. The CeO_2_ NP maintained negative zeta potential in both water and 1% FBS DMEM (pH 7.4), and as such, it can promote interaction with positively charged macromolecules in cell membranes to allow stronger NP attachment to cell surfaces, which results in more NPs being internalised into cells [35].

Central to the intent of the study, we found Col-I and α-SMA expression were reduced after CeO_2_ NP treatment, indicating that CeO_2_ NP were able to inhibit fibrogenesis, thereby decreasing the amount of secreted ECM material. This crucial early result demonstrated the pharmacological potential of CeO_2_ NP, rendering it purposeful to further investigate other fibrosis characteristics in CeO_2_ NP-treated cells.

Of interest, the regulation of the extracellular matrix microenvironment is not simply governed by the deposition of new collagen, but is also maintained by a counterbalance of fibrolytic activities. Under normal conditions, ECM content is regulated in part by quiescent HSCs releasing MMPs to actively degrade ECM material and prevent the excessive accumulation of ECM. In the presence of stress signals, activated HSCs posture to increase the amount of ECM through secreting ECM components, such as connective tissue proteins, and releasing TIMPs. Here, we found that MMP1 expression was upregulated while TIMP2 expression was downregulated in CeO_2_ NP-treated cells. MMP1 actively degrades ECM material, and MMP1 upregulation has been shown to reduce hepatic ECM accumulation [36]. TIMP2 inhibits MMP activity [37], and lowered TIMP2 expression similarly results in decreased ECM retention. Overall, CeO_2_ NP-treated cells demonstrated a dual-prong recalibration of ECM homeostasis towards a more quiescent cell-like manner of matrix regulation, supporting that CeO_2_ NP treatment reduces HSC activation. The concurrent modulation of both fibrogenesis and fibrolysis events offer a more robust control ECM maintenance to achieve therapeutic gains.

The fibrotic phenotype goes beyond the regulation of ECM dynamics and also includes morphological and mobility changes. Activated HSCs undergo epithelial-to-mesenchymal transitions (EMT), gain contractility and motility and migrate to wound sites where they secrete ECM material as part of the wound-healing response [1]. We found that CeO_2_ NP-treated cells had reduced motility when measured through transwell assays. They also had high E-cad and low N-cad expression, tending away from a mesenchymal predominance. The significantly diminished motility observed in 500 µM CeO_2_ NP-treated cells, together with the significantly less contractile phenotype compared to TGF-β controls, promulgate a quiescent HSC phenotype that limits further profibrotic activities and buys time for the liver at large to recover through removal of injury and regeneration of hepatocytes.

Fibrotic HSCs also display changes in their cellular maintenance. Autophagy is a cellular housekeeping machinery where unrequired cell components are degraded into their component molecules and recycled. HSC activation results in increased autophagy as HSCs undergo massive internal remodelling to complement their shift to a more mobile phenotype [38]. LC3 is a protein component of autophagosomal membranes and is commonly used for profiling autophagic flux [39,40]. The cargo adaptor protein p62 interacts with autophagic substrates and delivers them to autophagosomes for degradation. Thus, expression of p62 is inversely correlated with autophagic flux [41] and serves as a useful second confirmation for total autophagic activity. We found LC3 autophagic flux to be decreased in a dose-dependent manner, while p62 expression was increased, indicating that these cells demonstrated potentially reduced autophagy compared to TGF-β-activated controls. Coupling this with a lack of apoptosis, CeO_2_ NP are effective in inactivating HSCs without killing them, thereby desensitizing them to further TGF-β signals as a therapeutic advantage.

To address the molecular mechanism of CeO_2_ NP action on HSC, its antioxidant potential was investigated. The importance of oxidative stress in liver fibrosis is emphasised by studies identifying antioxidants as antifibrotic agents [42,43,44,45]. Crucially, we found that CeO_2_ NP treatment decreased oxidative stress in activated HSC cells, congruent with its known antioxidant properties. The 100 µM and 500 µM CeO_2_ NP treatments suppressed ROS release in a dose-dependent manner. We confirmed this through examining the expression of cellular antioxidant response genes. Nrf2 is a major regulator controlling cellular response to oxidative stress, and Nrf2 activity is often used as a surrogate for total cellular oxidative stress [46]. Total Nrf2 activity was significantly reduced in both CeO_2_ NP concentrations, suggesting an alleviation of the oxidative stress response as a trigger point. To confirm that Nrf2 activity was truly decreased in CeO_2_ NP-treated cells, we also studied the expression of the Nrf2 responsive gene NQO1 [47]. NQO1 expression was similarly found to be reduced, supporting our finding that Nrf2 activity was decreased in CeO_2_ NP-treated cells. The effect of CeO_2_ NP on downregulating the Nrf2 pathway has been demonstrated independently. CeO_2_ NP reduced oxidative stress levels and lowered Nrf2 and heme oxygenase-1 (HO-1) expression after 25 nm cubic CeO_2_ NP were used to alleviate D-galactosamine and lipopolysaccharide (D-GALN/LPS)-induced hepatotoxicity [48]. A human epithelial lung cell line also showed reduced Nrf2-responsive genes HO-1 and superoxide dismutase 2 (Sod2) expression after 25 nm cubic CeO_2_ NP pre-exposure [49]. CeO_2_ NP (4–20 nm spherical) also protected HepG2 hepatocytes from oxidative stress damage, partly through alteration of oxidative stress-related, kinase-signalling pathways [50]. Taken together, these results confirm that Nrf2 activity, and ultimately oxidative stress levels, were reduced in CeO_2_ NP-treated cells.

The alleviation of oxidative stress asserts a direct impact on the master regulator of hepatic fibrosis, i.e., TGF-β. Latent TGF-β is released by Kupffer cells and other immune cells in the form of an inactive complex comprising mature TGF-β, latency-associated protein (LAP) and latent TGF-β binding protein (LTBP) [51]. After activation, TGF-β is released from this complex and is then free to bind to TGF-β receptors on the surface of quiescent HSCs, initiating the Smad-signalling cascade and commencing HSC activation. Several studies have pointed to TGF-β signalling being sensitive to oxidative stress levels [42,51]. The dissociation of TGF-β from its latent complex requires several factors, including ROS signalling [51]. Oxidants can act on the latent TGF-β complex directly by oxidising LAP, dissociating it from the complex and releasing TGF-β, or indirectly through activating MMPs, which then cleave the complex and release TGF-β. Furthermore, active Smad signalling represses the expression of antioxidants, such as glutathione and Sod [51], and increases oxidative stress by upregulating ROS-generating enzymes, such as mitochondrial NADPH oxidases (NOX) [52,53]. Oxidative stress therefore activates HSCs partly through its effects on TGF-β signalling.

Agreeably, the hallmarks of TGF-β signalling, including Smad expression, were downregulated in CeO_2_ NP-treated cells through reduced levels of phosphorylated Smad2/3 and Smad4 levels. The question remains if the observed decrease in TGF-β signalling stems exclusively from the suppression of oxidative stress after CeO_2_ NP redox activity or is due to other extraneous factors not examined. CeO_2_ NP may have other intrinsic nonantioxidant properties that are able to reduce TGF-β signalling [54]. Future work should look to further tease apart this situation, possibly through the use of CeO_2_ NP treated to inhibit their redox capacity.

Our findings corroborated the observations of Oró and colleagues, when they treated CCl_4_-induced fibrotic rats with 4–20 nm spherical CeO_2_ NP [27]. However, they found that CeO_2_ NP-treated rats showed reduced expression of α-SMA, but not Col-I. This was a surprising finding, as Col-I is a marker for ECM and fibrosis, and suggests that overall fibrosis levels were not reduced in these animals. Similarly, Hirst et al. treated mice with CCl_4_-induced fibrosis with CeO_2_ NP and found reduced levels of lipid peroxidation as measured through MDA levels [19]. Unfortunately, they did not report on the degree of fibrosis observed, and hence the status of fibrosis in these mice is unknown.

We reason that this deviation from our findings may be due to the amount of CeO_2_ NP that enter HSCs in vivo not reaching sufficiently high enough concentrations required for therapeutic benefit. Our study indicated that relatively high concentrations of CeO_2_ NP were required to significantly inhibit fibrosis symptoms (100 µM, or 17.2 mg/L; and 500 µM, or 86.1 mg/L). To give biological context to this in vitro dosage, Yokel et al. injected a similar concentration of 85 mg/kg CeO_2_ NP (30 nm, cubic) into rats and reported that the liver retained 4.6 mg and 4.43 mg of total cerium 30 and 90 days post-injection respectively [17]. Oró et al. injected fibrotic rats with 0.1 mg/kg CeO_2_ NP twice weekly for two weeks. They then used inductively coupled plasma-mass spectroscopy (ICP-MS) to quantify the amount of total cerium in rat livers after eight weeks post-injection and found that these livers accumulated approximately 15 µg/g of total cerium [27]. This is less than our low dose (17.2 mg/L) found to inhibit fibrogenesis in our study. Furthermore, after entry into the liver, CeO_2_ NP are predominately taken up by Kupffer cells and hepatocytes, with Kupffer cells responsible for phagocytosing the large majority of NP [19,21,22,55]. This suggests that HSC NP uptake is comparatively little compared to macrophages and hepatocytes, and thus HSCS may only accumulate a small fraction of the 15 µg/g of total liver cerium from Oró et al. Tseng et al. investigated CeO_2_ NP accumulation (30 nm, cubic) in various liver cell types at the ultrastructural level [22]. From their presented TEM images, it is noticeable that the amount of CeO_2_ NP shown in an HSC cell after 90 days post-exposure was lower when compared to adjacent images of a Kupffer cell and a hepatocyte. We therefore suspect that the amount of CeO_2_ NP entering HSCs in previous studies may have been insufficient to completely treat hepatic fibrosis in vivo.

To address this limitation in a physiologically complete system, HSC-specific targeting mechanisms can be added to CeO_2_ NP to directly target them to HSCs and to avoid being taken up by other liver cell types. Adding an HSC-specific sequence to directly deliver CeO_2_ NP to HSCs may increase HSC CeO_2_ NP in vivo accumulation to levels approaching therapeutic benefits. Several methods have been proposed for use as HSC targeting sequences [56]. HSC-targeting CeO_2_ NP can also be loaded with cytotoxic drugs such as sunitinib, which will kill activated HSCs, reducing the number of activated HSCs and thereby reducing liver fibrosis levels in vivo. As proof of this concept, Sulthana et al. conjugated folic acid onto the surface of CeO_2_ NP carrying doxorubicin and the HSP90 inhibitor ganetespib, which allowed efficient targeting of non-small-cell lung cancer cells that overexpressed folic-acid receptor [57]. This resulted in vastly increased killing efficiency compared to nonfolic-acid-conjugated drug-loaded CeO_2_ NP after 48 h. This report demonstrates that CeO_2_ NP can act as efficient drug carriers and suggests a future technique where modifying CeO_2_ NP with an HSC-targeting signal will allow effective targeting of HSCs in vivo.

Additionally, CeO_2_ NP can also be surface modified with protective coatings, such as polyethylene glycol (PEG) or albumin. These coatings have been shown to reduce degradation by body components after introduction, thereby increasing the amount of NPs reaching target organs [58]. PEG coatings have even been shown to reduce NP phagocytosis by Kupffer cells [59], further increasing the amounts reaching HSCs. Importantly, PEG-coated CeO_2_ NP still retain their antioxidant capabilities [60], which suggests that surface modifying CeO_2_ NP with a PEG layer will not hinder their antifibrotic effects.

Overall, while metallic NP are often categorically dismissed as being detrimental for health, our work underscores the reality that the actual situation may be more nuanced, as each element has its own intrinsic properties that can dictate their specific interactions with biological systems [5]. While our study and numerous others have found that CeO_2_ NP can have therapeutic potential for liver diseases, their role in the lung is considerably less beneficial, with CeO_2_ NP inhalation being found to induce pulmonary inflammation and fibrosis in rats [61,62]. It is therefore crucial to thoroughly investigate the effects of each NP carefully in the correct biological context before deciding if a particular NP type is beneficial or detrimental to a biological system. Along this line, future work can include examining the effects of exposing quiescent LX2 cells to CeO_2_ NP to understand the effects of CeO_2_ NP on fibrosis induction.

Our study demonstrated that CeO_2_ NP were able to reduce hepatic fibrosis symptom levels in vitro. This was achieved through lowering oxidative stress and TGF-β signalling, ultimately reducing the activation of CeO_2_ NP-treated LX2 cells. Our work indicated that contrary to previously published reports, the final conclusion for the effect of CeO_2_ NP on liver fibrosis is still unclear, and CeO_2_ NP may yet be a potential therapy for hepatic fibrosis.

## 4. Materials and Methods

### 4.1. Nanoparticles Used in This Study

CeO_2_ NP (catalogue number 544841) and TiO_2_ NP (catalogue number 718467) were purchased from Sigma-Aldrich (St. Louis, MO, USA). The size, shape and charge of TiO_2_ NP was described previously [31]. NPs were freshly prepared for each experiment. NP powder was weighed to appropriate amounts to make a stock solution, dissolved in ultrapure water and dispersed using probe sonication (Fisher Scientific, Pittsburgh, PA, USA) for 1 min to minimise NP aggregation and ensure a disperse single particle suspension. This dispersed solution was then added to media at appropriate concentrations before the media-NP solution was probe sonicated again for 1 min just before use. CeO_2_ NP were first characterised for their size and shape using transmission electron microscopy. 10 µL of 100 µM CeO_2_ NP solution was pipetted directly onto formvar-coated grids and incubated for 1 min, before being washed with distilled water, dried and imaged on a Tecnai G^2^ Spirit operating at 120 kV (FEI, Hillsboro, OR, USA).

### 4.2. Cell Lines Used

The human hepatic stellate cell LX2 (received as a kind gift from Professor Scott Friedman) was used in this study. LX2 cells were maintained in Dulbecco’s Modified Eagle’s Medium (Sigma-Aldrich, USA) containing 10% FBS (Gibco, Amarillo, TX, USA) supplemented with penicillin-streptomycin (Gibco, USA) at 37 °C and 5% CO_2_. One generation prior to use in experiments, cells were subcultured in 1% FBS DMEM to ensure quiescence. LX2 cells were activated using 2 ng/mL TGF-β (STEMCELL Technologies, Vancouver, BC, Canada) in 1% FBS DMEM.

### 4.3. Dynamic Light Scattering

Before analysis, 100 µM CeO_2_ NP was resuspended in 1 mL of ultrapure water or 1% FBS DMEM. Hydrodynamic size and zeta potential were measured using a Litesizer 500 (Anton Paar, Graz, Austria).

### 4.4. MTS Assay

Cell viability was measured using MTS assay (CellTiter 96^®^ AQueous One Solution Cell Proliferation Assay, Promega, Madison, WI, USA). Overnight, 10,000 cells per well were seeded before being treated with 2 ng/mL TGF-β and appropriate NPs for 24 h. Each treatment was performed in triplicate. An hour before the incubation period finished, 20 µL of MTS reagent was added per well and incubated for an hour at 37 °C before being read on a plate reader (Tecan, Männedorf, Switzerland) at 490 nm.

### 4.5. Cell Imaging with Light Microscopy

Overnight, 200,000 cells were seeded in six-well plates before being treated for 24 h with 2 ng/mL TGF-β and appropriate NPs. Cells were washed in PBS, before images taken at 10× magnification with an Olympus DP21 camera (Olympus, Shinjuku, Tokyo, Japan).

### 4.6. Cell Migration Assay

Overnight, 200,000 cells were seeded in six-well plates before being treated for 24 h with 2 ng/mL TGF-β and appropriate NPs. After the treatment period, cells were then stained with 1 µg/mL CellTracker Orange CMRA Dye (Invitrogen, Waltham, MA, USA) for 45 min at 37 °C. Cells were then removed from the plate, counted and seeded into the inner chamber of a transwell insert (8 µm pore size, Corning Costar, Cambridge, MA, USA) at 10,000 cells/insert in 100 µL of serum-free DMEM. The insert was then carefully lowered into a 24-well plate containing 600 µL of 10% FBS DMEM. Cells were then incubated at 37 °C for a further 24 h. A cotton bud was then used to carefully remove media and cells from the upper membrane layer inside the insert before the membrane was fixed with 10% formalin (Sigma-Aldrich, St. Louis, MO, USA). Cells were then stained with 2 µg/mL Hoechst 33342 stain for 30 min at room temperature. The transwell insert membrane was then carefully removed and mounted on a glass slide before imaging using an Olympus FV100 confocal microscope (Olympus, Shinjuku, Tokyo, Japan). At least 16 random images were taken per sample at 20× magnification, and the total number of cells were counted per sample and normalised to TGF-β controls.

### 4.7. Oxidative Stress Measurement

Overnight, 600,000 LX2 cells were seeded in a 60 mm dish and treated with 2 ng/mL TGF-β and appropriate NPs for 24 h. Samples were then washed with PBS and incubated with 5 µM CellROX Deep Red (Thermo Fisher Scientific, Waltham, MA, USA) for 30 min at 37 °C. Cells were then washed with PBS and treated with trypsin (Hyclone, Logan, UT, USA) for 5 min at 37 °C. Serum-free DMEM was then used to inactivate the trypsin. Cells were then washed again with PBS before being fixed with 10% formalin for 15 min at room temperature. Cells were then washed with PBS, then resuspended in 250 µL of PBS before being analysed on a CytoFLEX Platform flow cytometer (Beckman Coulter, Brea, CA, USA). We used 20,000 events per sample to calculate mean fluorescence intensity per cell and ultimately ROS accumulation per sample and normalised to TGF-β controls.

### 4.8. Nrf2 Activity Assay

The ARE Reporter Kit (BPS Bioscience, San Diego, CA, USA) was used to study total Nrf2 activity according to manufacturer’s instructions. Briefly, 10,000 LX2 cells were seeded overnight in a white, clear-bottom, 96-well plate (NUNC, Rochester, NY, USA), then transfected with ARE Reporter plasmid or Negative Control Reporter plasmid using lipofectamine 2000 (Invitrogen, Waltham, MA, USA). Cells were then left to recover for 24 h at 37 °C. Cells were then treated with 2 ng/mL TGF-β and appropriate NPs for 24 h before luciferase activity was measured using a Dual-Luciferase Reporter Assay System (St. Louis, MO, Promega, USA) on a 96-well plate reader (Tecan, Männedorf, Switzerland) and data normalised to TGF-β controls.

### 4.9. Immunoblots

Overnight, 600,000 LX2 cells were seeded in a 60 mm dish and treated with 2 ng/mL TGF-β and appropriate NPs for 24 h. Total cell proteins were extracted with radioimmunoprecipitation assay (RIPA) buffer supplemented with protease inhibitors aprotinin, sodium orthovanadate, sodium fluoride and phenylmethylsulfonyl fluoride (PMSF). For blots investigating autophagic flux, chloroquine was used to inhibit autophagy to permit the calculation of autophagic flux. Treatment media was removed 4 h before the incubation period ended, and replaced with fresh media containing 40 µM of chloroquine, before total cell proteins were extracted as above. Protein concentration was determined using the Pierce BCA Protein Assay Kit (Thermo Fisher Scientific, Waltham, MA, USA) according to the manufacturer’s instructions. We loaded 20–30 µg of protein per sample on 10% or 15% SDS-polyacrylamide gels (PAGE). Samples for denaturing gels were mixed with 6X SDS loading dye and boiled at 95 °C for 5 min before loading. Samples for native gels were mixed with native sample buffer (Bio-Rad, Hercules, CA, USA) and loaded. Gels were run at 100 V before being transferred to polyvinylidene fluoride (PVDF) membranes using a wet transfer system (Bio-Rad, Hercules, CA, USA). Membranes were then blocked with 5% BSA in tris-borate saline buffer with 0.1% tween-20 before being probed overnight at 4 °C with primary antibodies, and subsequently probed with the appropriate species-specific horseradish peroxidase (HRP)-conjugated secondary antibody for 1 h at room temperature. Blots were developed with Pierce ECL Western Blotting Substrate (Thermo Fisher Scientific, Waltham, MA, USA) and imaged on a G:BOX Chemi XX6 system (Syngene, Bangalore, India). Relative band intensities to GAPDH were calculated using ImageJ [63] and plotted using Graphpad Prism (San Diego, California, USA).

The antibodies used in this study include anti-GAPDH antibody (#5174, Cell Signaling Technology, Danvers, MA, USA), anti-phos-Smad2/3 (#8828S, Cell Signaling Technology, Danvers, MA, USA), anti-Smad2/3 (#8685S, Cell Signaling Technology, USA), anti-Smad4 (#38454S, Cell Signaling Technology, USA), anti-collagen I (ab34710, ABCAM, Cambridge, UK), anti-Smooth Muscle Actin (M0851, Dako, Glostrup, Denmark), anti-microtubule-associated proteins 1A/1B light chain 3B (LC3B) (#2775S, Cell Signaling Technology, Danvers, MA, USA), anti-p62 (ab56416, ABCAM, Cambridge, UK), anti-NQO1 (#62262, Cell Signaling Technology, Danvers, MA, USA). Our phosphorylated Smad2/3 antibody recognises the Ser465/467 site on Smad2 and the Ser423/425 site on Smad3.

### 4.10. RNA Extraction, cDNA Synthesis and Real-Time PCR

Overnight, 200,000 LX2 cells per well were seeded in six-well plates and treated with 2 ng/mL TGF-β and appropriate NPs for 24 h. RNA was extracted using trizol (Invitrogen, Waltham, MA, USA), according to manufacturer’s instructions. We used 750 ng of RNA per sample to synthesise complementary DNA (cDNA) using Superscript II (Invitrogen, USA). Expression levels of target genes were determined using Green-2-Go qPCR mix (BioBasic, Toronto, Canada) on a CFX96 real-time PCR machine (Bio-Rad, Hercules, CA, USA). CT values were calculated using the CFX96 software. Gene expression was normalised to glyceraldehyde-3-phosphate dehydrogenase (GAPDH) mRNA expression using the comparative ΔΔC_t_ method and plotted using Graphpad Prism (San Diego, CA, USA). The following primers were used: GAPDH: 5′-ACTTTGGTATCGTGGAAGGACT-3 (forward) and 5-GTAGAGGCAGGGATGATGTTCT-3′ (reverse) [31]; Col-I: 5′-CCTGGATGCCATCAAAGTCT-3′ (forward) and 5-CGCCATACTCGAACTGGAAT-3′ (reverse) [31]; α-SMA: 5-CCGGGAGAAAATGACTCAAA-3′ (forward) and 5-GCAAGGCATAGCCCTCATAG-3′ (reverse) [31]; E-cad: 5- AATTCCTGCCATTCTGGGGA-3′ (forward) and 5′- TCTTCTCCGCCTCCTTCTTC-3′ (reverse) [31]; and N-cad: 5′-TGAGCCTGAAGCCAACCTTA-3′ (forward) and 5′-AGGTCCCCTGGAGTTTTCTG-3′ (reverse) [31]; MMP1: 5′-CAGAGATGAAGTCCGGTTTTTC-3′ (forward) and 5′-GGGGTATCCGTGTAGCACAT-3′ (reverse) [64]; TIMP2: 5′-GAGCCTGAACCACAGGTACCA-3′ (forward) and 5′-TCTGTGACCCAGTCCATCCA-3′ (reverse) [65].

### 4.11. Caspase 3/7 Activity Assay

Caspase 3/7 activity was determined using the Caspase 3/7-GLO assay kit (Promega, Madison, WI, USA). Overnight, 10,000 cells were seeded in a white, 96-well plate and treated with 2 ng/mL TGF-β and appropriate NPs for 24 h. Each treatment was performed in triplicate. Cells were subsequently treated with Caspase 3/7-GLO assay reagents according to manufacturer’s instructions. Luminescence was measured on a microplate reader (Tecan, Männedorf, Switzerland) and data normalised to TGF-β controls.

### 4.12. Statistical Analysis

All experiments were performed in at least three replicates (*n* = 3). One-way ANOVA followed by Dunnett’s multiple comparisons test was performed using GraphPad Prism version 8.0.0 for Windows, GraphPad Software (San Diego, CA, USA), www.graphpad.com accessed on 11 February 2021. Error bars represent standard error of the mean. Statistical significance was set at *p* ≤ 0.05.

## Figures and Tables

**Table 1:** ROS-level reduction in CeO_2_-treated cells.

Sample	Mean ROS Level	Difference from TGF-β-activated Cell Mean *	Percentage Reduction from Quiescent Cell Mean ^#^
Quiescent cell	0.817	−0.183	N.A.
TGF-β-activated cell	1.00	N.A.	N.A.
100 µM CeO_2_ NP	0.936	−0.064	35.03%
500 µM CeO_2_ NP	0.793	−0.207	113.0%
100 µM TiO_2_ NP	1.04	+0.40	−26.20%

* (TGF-β-activated cell mean ROS level)-(NP sample mean ROS level). # $\frac{\left(\mathrm{TGF}-\beta -\mathrm{activated}\;\mathrm{cell}\;\mathrm{mean}\;\mathrm{ROS}\;\mathrm{level}\right)-\left(\mathrm{NP}\;\mathrm{sample}\;\mathrm{mean}\;\mathrm{ROS}\;\mathrm{level}\right)}{\left(\mathrm{TGF}-\beta -\mathrm{activated}\;\mathrm{cell}\;\mathrm{mean}\;\mathrm{ROS}\;\mathrm{level}\right)-\left(\mathrm{Quiescent}\;\mathrm{cell}\;\mathrm{mean}\;\mathrm{ROS}\;\mathrm{level}\right)}\times 100$.

## Data Availability

Not applicable.

## Supplementary Materials

The following are available online at https://www.mdpi.com/article/10.3390/ijms222111777/s1.
