# Supplementary material for: Cerium Oxide Nanoparticles Alleviate Hepatic Fibrosis Phenotypes In Vitro
Source: Int J Mol Sci. 2021 Oct 29;22(21):11777. doi: 10.3390/ijms222111777 (PMC8584085; doi:10.3390/ijms222111777)
Supplement: Supplementary file 1 [file ijms-22-11777-s001.zip › ijms-1369682-supplementary.pptx]

## Slide 1
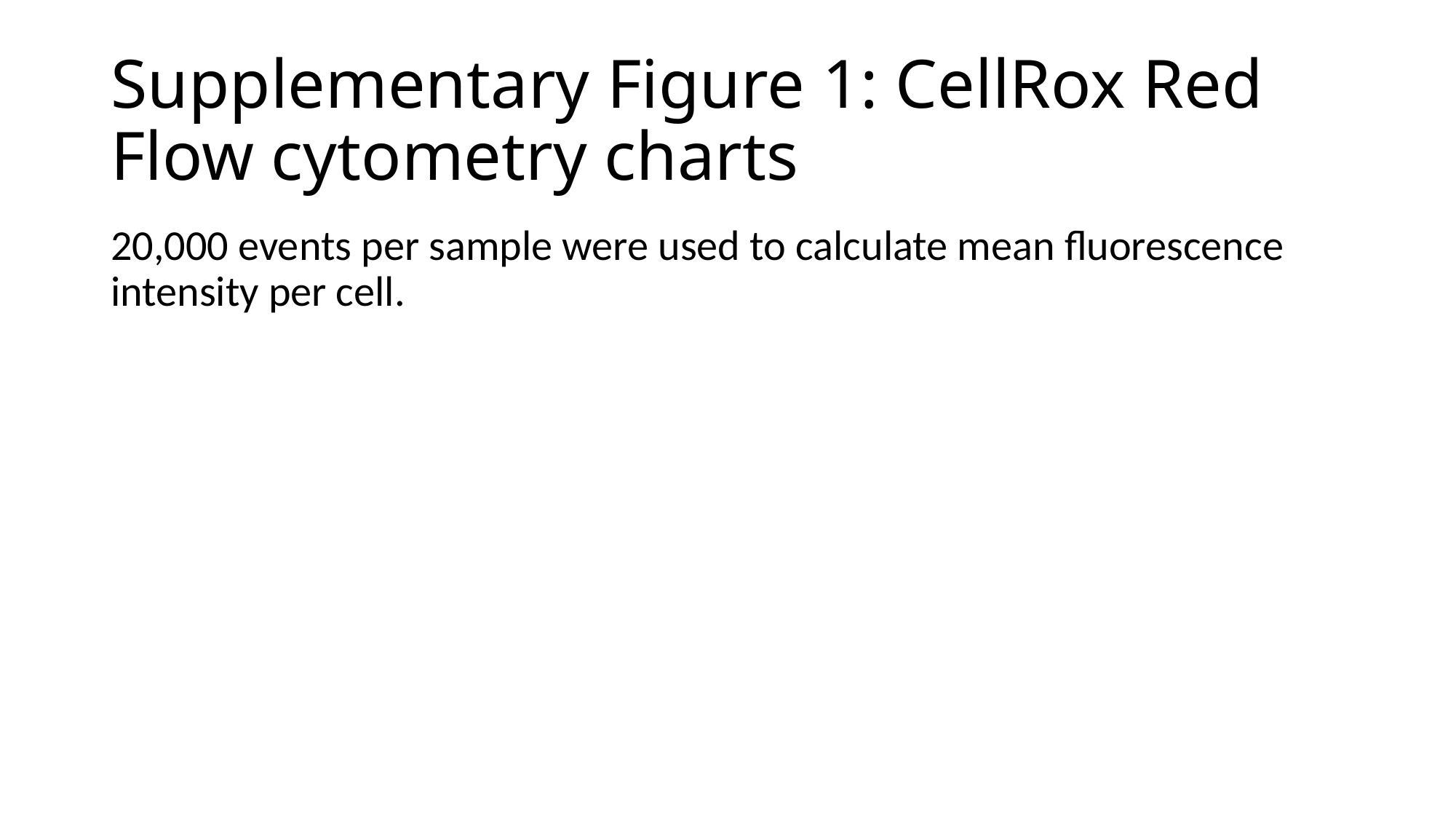

# Supplementary Figure 1: CellRox Red Flow cytometry charts
20,000 events per sample were used to calculate mean fluorescence intensity per cell.

## Slide 2
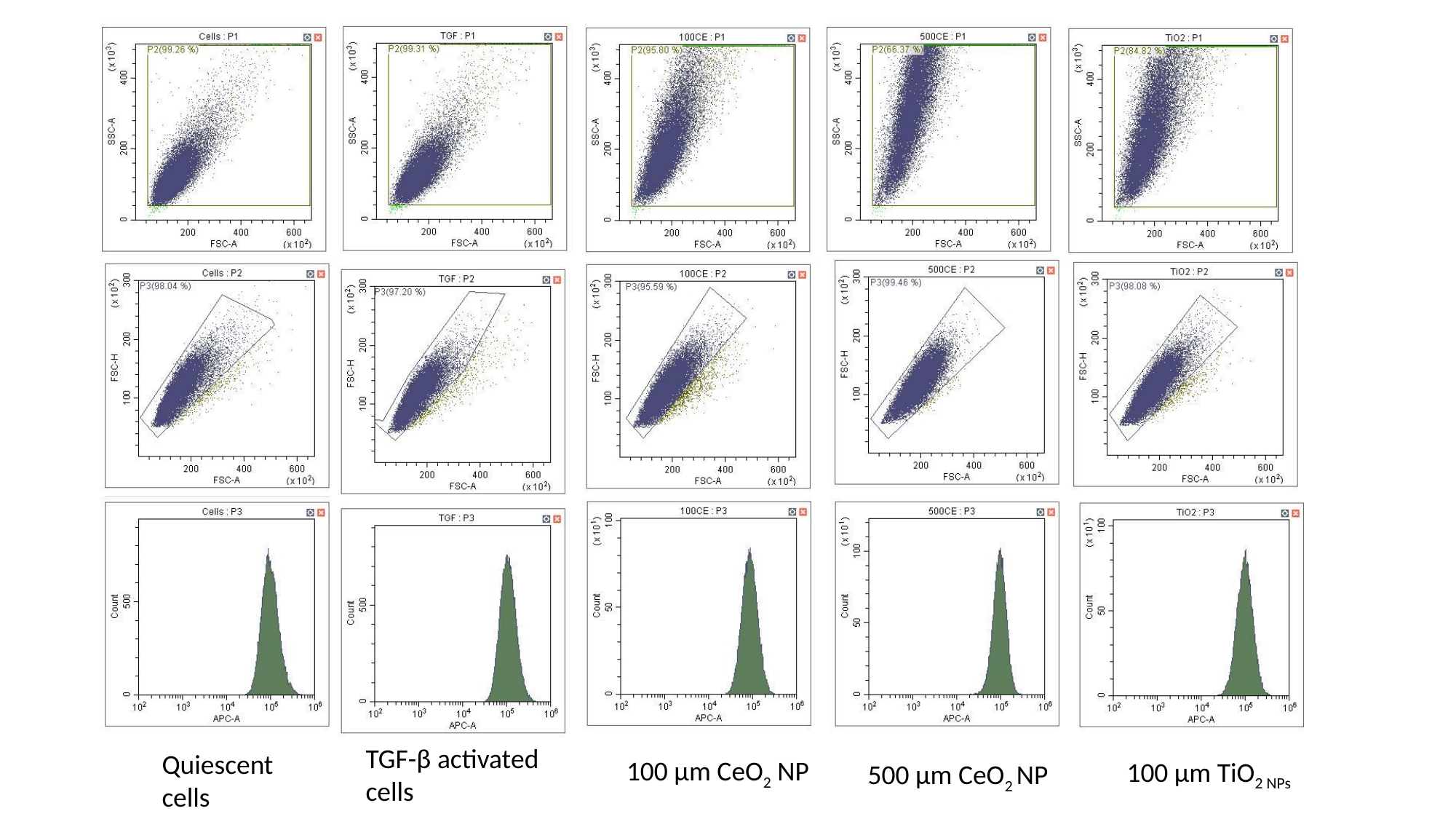

TGF-β activated
cells
Quiescent cells
100 µm CeO2 NP
100 µm TiO2 NPs
500 µm CeO2 NP
